# A reservoir-damage-free encapsulated acid dually controlled by hydrogen ion concentration and temperature

**DOI:** 10.1039/c9ra06763a

**Published:** 2019-10-21

**Authors:** Zhifeng Luo, Nanlin Zhang, Liqiang Zhao, Lin Wu, Pingli Liu, Dengfeng Ren, Chun Qing

**Affiliations:** State Key Laboratory of Oil and Gas Reservoir Geology and Exploitation, Southwest Petroleum University Chengdu 610500 P. R. China nanlin_zhang@163.com zhaolq@163.vip.com; PetroChina Tarim Oil Company Korla 841000 P. R. China; Southwest Oil & Gas Field Company, CNPC Chengdu 610500 China

## Abstract

Oil and gas exploration and development extends from medium-low temperatures to high and ultra-high temperatures with the development of the oil and gas industry. High-temperature deep carbonate reservoir acid fracturing has introduced more stringent requirements, a slower chemical reaction rate and excellent dissolution performance of acid systems, which means that the acid system should still have a certain dissolution ability above 135 °C. A novel water-soluble encapsulated acid (EA), dual controlled by hydrogen ion concentration and temperature, was developed to exploit ultra-high-temperature carbonate reservoirs. The encapsulating material was insoluble and isolated the internal solid acid at high H^+^ ion concentrations and low temperatures, but the solid acid was released as the encapsulating material was dissolved at low H^+^ ion concentrations and high temperatures. This unique performance was characterized by ESEM, TGA, FTIR, NMR, mechanical performance, solubility, etching performance, and etching fracture conductivity. All the scientific results show that this EA can be applied as a long-distance etching acid controlled by H^+^ ion concentration and temperature, without the need for a thickener and emulsifier to reduce the reaction between the rock and the acid near the wellbore. The test results demonstrated that the solid acid had good thermal stability at 135 °C, the encapsulation material was almost insoluble in high acid concentrations (>14%) at any temperature, and the solid acid began to release when the concentration of hydrochloric acid was less than 14% and the temperature was higher than 95 °C. The rock etching and dissolution behavior was better than that of HCl with the same concentration and the etching fracture conductivity was improved by supplementing the consumption of H^+^ ions when etching rock. The encapsulating material is completely dissolved after acid fracturing, avoiding reservoir damage by the residue. The described EA is a promising approach for application in acid fracturing of carbonate reservoirs at ultra-high temperature (>135 °C).

## Abstract 2

Oil and gas exploration and development extends from medium-low temperatures to high and ultra-high temperatures with the development of the oil and gas industry.

## Graphical abstract

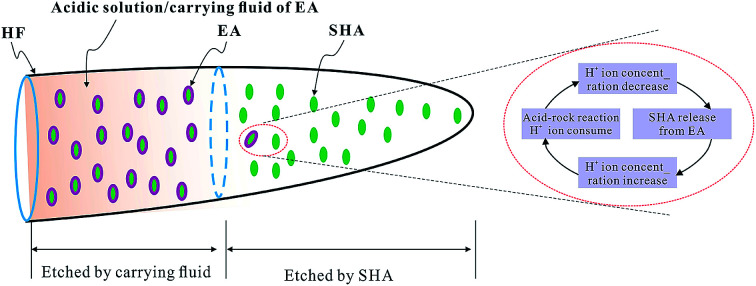

## 1. Introduction

With the development of the global oil and gas industry, oil and gas exploration and development extends from shallow layers to deep layers and ultra-deep layers and changes rapidly from medium-low temperatures to high and ultra-high temperatures.^1–3^ High-temperature deep carbonate reservoir acid fracturing has introduced more stringent requirements,^4–6^ a slower chemical reaction rate and excellent dissolution performance of acid systems, which means that the acid system still should have a certain dissolution ability above 135 °C.^7–9^ Existing acids for high temperatures with a slow chemical reaction rate include emulsified acids,^10,11^ organic acids^12,13^ and cross-linked acids.^14^ Although these acid systems have a certain slow chemical reaction rate performance, they are in direct contact with the acid fracturing pipelines and therefore must be treated with corrosion inhibitor additives.^15–17^ The concept of encapsulated acids, which use encapsulating materials to cover solid hydrochloric acid,^18^ nitric acid^19^ and citric acid,^20–22^ has been researched, as has the isolation of solid acids by means of encapsulation methods. The penetration depth of acids has been increased by the temperature and time-controlled dissolution performance of different encapsulating materials. Although EAs can solve the problem of corrosion, they are limited by drawbacks such as the low effective H^+^ ion concentration of acids, poor thermal resistance, and the short penetration depth of the acids. According to previous studies indicating that a water-insoluble EA^23^ may cause formation damage owing to the insoluble matter, improvement of the thermal resistance, mechanical strength, toughness and solubility of encapsulating materials can enhance the penetration of the EA and avoid the formation damage. In the present study, the tetrapolymer used as the encapsulating material, known as the H^+^ ion-temperature responsive coating material (HTRCM), was prepared by copolymerization of acrylamide monomers, *tert*-butyl allyl carboxylate monomers, diallylamine monomers, and allyl alkyl polyether monomers. The solid hydrochloric acid was encapsulated to prepare the EA. Therein, acrylamide, *tert*-butyl allyl carboxylate, diallylamine, and allyl alkyl polyether were used as backbone monomers, acid-resistant monomers, strength monomers, and toughness monomers, respectively.

**Fig. 1:**
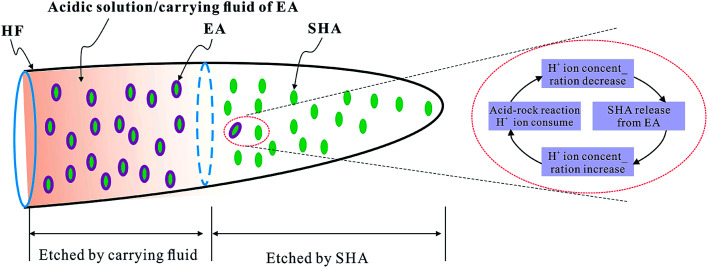
The dynamic process of the H^+^ ion concentration in the acidic solution and EA migration to the far end of the fracture (HF: hydraulic fracture, EA: encapsulated acid, SHA: solid hydrochloric acid).

The proposed EA was carried by the acidic solution. The acidic solution has two functions; carrying the EA and also etching the surface of the rock in the near-well region. Unlike other EAs,^18–22^ the release of the proposed EA is dual controlled by the H^+^ ion concentration and the temperature. With low temperatures (<95 °C) and high H^+^ ion concentrations of the carrying fluid (>14%), the encapsulating material should maintain its structural integrity, isolate the carrying fluid from the solid hydrochloric acid (SHA), and prevent the solid hydrochloric acid from being dissolved. With the increase of the temperature (>95 °C) and a decrease in the H^+^ ion concentration (<14%), the encapsulating material dissolves and releases the solid hydrochloric acid. Ionization produces H^+^ ions, meaning that the H^+^ ion concentration of the acidic solution is restored. During this process, the H^+^ ion concentration undergoes a dynamic change process, as illustrated in Fig. 1:

The above characteristics of the proposed EA made good use of the hydraulic fracture with the low temperature and high H^+^ ion concentration in the near-well region as well as at the high temperature and low H^+^ ion concentration in the far-well region.^24,25^ In the near-well region, H^+^ ions of the acidic solution are used to etch the fracture surface without releasing the SHA. In the far-well region, the SHA is released and etches the fracture faces, effectively increasing the penetration depth of the acid. The carrying fluid etches the fracture faces in the near-well region^26^ and the EA etches the fracture faces in the far-well region, effectively increasing the effective penetration distance of acid fracturing, as shown in Fig. 1. Therefore, the proposed EA is of great potential significance for the acid fracturing reconstruction of high-temperature deep reservoirs.

## 2. Methodology

### 2.1. Materials

Hydrazine hydrate (N_2_H_4_·H_2_O), hydrochloric acid (HCl), acrylamide monomers (AL), *tert*-butyl allyl carboxylate monomers (AC), diallylamine monomers (DL), allyl alkyl polyether monomers (AAP), absolute ethanol (AE), anion polyacrylamide (AP), ethyl acetate (EAC) and dichloromethane (DE) were purchased from Kelong in Chengdu. All chemicals and reagents were utilized without further purification.

### 2.2. Preparation of SHA, HTRCM, and EA

**Fig. 2:**
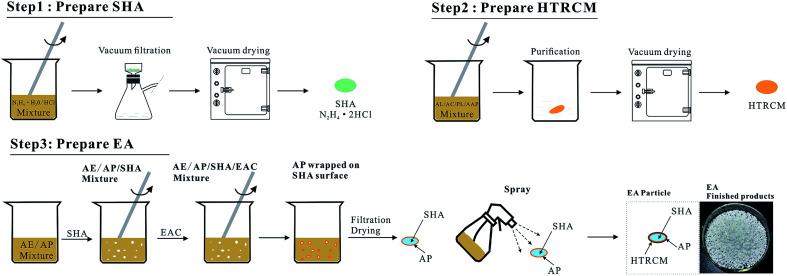
Flow chart of the preparation of SHA, HTRCM, and EA.

The preparation process was divided into three steps, comprising the preparation of SHA, the preparation of the encapsulating material HTRCM and the preparation of the EA. The process is illustrated in Fig. 2:

The first step was the preparation of the SHA. 10 mL of hydrazine hydrate (concentration: 80%) and 25 mL of concentrated hydrochloric acid (concentration: 36%) were placed in a beaker. After reaction at 15 °C for 45 minutes, suction filtration was applied to achieve solid–liquid separation at 20 °C with a suction filtration time of 10 min. The separated solid was then dried in a vacuum drying oven at 80 °C for 2 h. Finally, the prepared solid hydrochloric acid (N_2_H_4_·2HCl) was stored for use.

The second step was the preparation of the encapsulation material, HTRCM. 20 mL of AE, 4 g of AL, 5 g of AC, 0.5 g of PL, and 0.5 g of AAP were mixed in a beaker and stirred at 55 °C for 10 h. The mixture then was precipitated with AE. Then the prepared HTRCM encapsulating material was dried in a vacuum drier and stored for use.

The third step was the preparation of the EA. 10 mL of AP and 10 mL of AE mixture were stirred to complete dissolution in a beaker. The SHA prepared during the first step was added into the beaker and stirred at a constant rate. After adding EAC, AP precipitated and adhered to the surface of the SHA as a protective coating. Then the precipitated solid was filtered, dried at 80 °C for 24 h, and placed in a coating pan. The coating pan was rotated at a speed of 80 rpm and a temperature of 70 °C. HTRCM was mixed with AE to prepare a spray solution, which then was evenly sprayed on the solid surface to obtain the EA.

### 2.3. ESEM and energy spectrum analysis of EA

An FEI Quanta 450 environmental scanning electron microscope with an attached ESAX X-ray energy spectrometer was used to observe the micromorphology of the EA surface and analyze the elemental composition.

### 2.4. Thermogravimetric analysis of the SHA

The SHA described in this paper was a pure substance that was formed by replacing the water molecules in hydrazine hydrate with hydrochloric acid. Its thermogravimetric analysis was related to the parameters of the encapsulation material, the effective concentration of H^+^ ions, *etc.* It was necessary, therefore, to test the thermal stability of the SHA. A simultaneous thermal analyzer (NETZSCH STA449 F3) was used to test the weight change and heat change of the SHA in the range of 45–400 °C.

### 2.5. FTIR analysis of the HTRCM

KBr and HTRCM were mixed to uniformity at a mass ratio of KBr : HTRCM = 50 : 1 before being ground and compressed. The infrared spectrum was measured using a WQF-520A FTIR. The testing temperature was 25 °C and the number of scans was 16.

### 2.6. NMR analysis of the HTRCM

20 mg of the HTRCM sample and 1.00 mL of D_2_O were added to an EP tube. After the mixture had dissolved completely, 0.60 mL samples were taken to the 5 mm NMR tube. A Bruker Avance III 400 MHz NMR spectrometer was used to test the ^1^H NMR spectrum of the sample with the liquid BBO normal-phase observation broadband probe.

### 2.7. Mechanical performance test of the HTRCM

HTRCM was used as the encapsulating material for the EA, and when the EA was injected into the reservoir, it was affected by pressure, friction, *etc.* The HTRCM needs to have a certain film-forming ability and mechanical strength. Hence, the mechanical performance of the HTRCM was tested.

The material was dissolved in AE to prepare a 0.05 g mL^−1^ solution, which was poured into a polystyrene dish and placed in an oven at 35 °C, allowing the AE to evaporate to obtain a film formed by the HTRCM.

The film formed by the HTRCM was cut into samples of about 5.0 mm in width. The tensile strength and maximum load of the samples were measured using a CWT6104 electronic universal testing machine (Meters Industrial Systems Co., Ltd.). The samples were tested three times. The average tensile stress was taken as the final result.

### 2.8. Dissolution test of the HTRCM

The dissolution of the HTRCM at room temperature (25 °C) was studied using different polar solvents. Appropriate amounts of the HTRCM were accurately weighed and added into five 2.0 mL centrifuge tubes. Next, 1.5 mL of various solvents, which were AE, EAC, DE, deionized water, and NaOH solution (0.01 g mL^−1^), was added into the tubes. After storage at 25 °C for 24 h, the dissolution states of all materials were observed. All the solutions in the centrifuge tubes were filtered by a 150 mesh screen and the obtained residues were dried at 80 °C under vacuum for 2 days. The dried HTRCM was weighed and the material residual rate was calculated using eqn (1).1
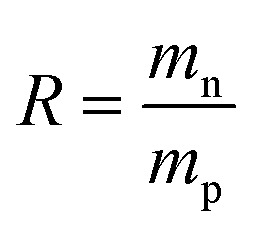
where *R* refers to the material residual rate and *m*_p_ and *m*_n_ refer to the mass of HTRCM before and after the dissolution experiment, respectively.

As the EA was carried by the acidic solution, the dissolution performance of the HTRCM in concentrated hydrochloric acid (mass fraction 37%) and sodium hydroxide solution (0.01 g mL^−1^) was investigated. The method used was: two appropriate amounts of HTRCM were weighed and put into two PTFE reactors with high-pressure reaction kettles. 15.00 mL samples of the concentrated hydrochloric acid and the sodium hydroxide solution were taken and added to the above PTFE reactors, and the reaction kettles were sealed. The reaction kettles were put into an electric blast drying oven at a constant temperature of 135 °C for 3 hours, after which the reaction kettles were taken out and cooled to room temperature. Then all the solutions in the reaction kettles were filtered using a 150 mesh screen and the obtained residue was dried at 80 °C under vacuum for 2 days. The dried HTRCM was weighed accurately and the material residual rate was calculated using eqn (1).

### 2.9. Effect of the H^+^ ion concentration in the carrying fluid on the release of EA

When the H^+^ ion concentration in the carrying solution was high, the HTRCM was insoluble and encapsulated the SHA. When the H^+^ ion concentration in the carrying fluid decreased, the HTRCM was gradually damaged and water in the carrying fluid penetrated into the EA through the HTRCM, dissolving and releasing SHA. The effect of the acid concentration on the SHA release was tested. The test method was as follows: (1) 20 mL samples of hydrochloric acid with concentrations of 0%, 1%, 2%, …, 28%, 29%, and 30% were added separately to the PTFE reactor. (2) EA with an accurate mass of *m*_p_ was put into the acid with different concentrations from step (1). (3) The PTFE reactor was heated at 135 °C for 1 hour and then taken out. It was filtered using a 150 mesh screen to obtain the solid. (4) The obtained solid was dried at 80 °C under vacuum until the solid weight was constant. (5) The obtained solid was weighed, the mass was recorded as *m*_n_ and the mass residual rate was calculated according to eqn (1).

### 2.10. Temperature influence on the SHA release

Temperature can affect the acid release rate by affecting the dissolution performance of the HTRCM in the carrying fluid. The effect of temperature on the rate of EA release was therefore investigated. The test method was as follows: (1) 20 mL samples of hydrochloric acid with concentrations of *c* (*c* = 11%, 12%, 13%, 14%, 15%, 16%) were added separately to the PTFE reactor. (2) EA with an accurate mass of *m*_p_ was put into the hydrochloric acid. (3) The PTFE reactor was heated in an electric blast drying oven separately at a constant temperature of *T* (where *T* = 85 °C, 95 °C, 105 °C, 115 °C, 125 °C, and 135 °C) for 1.0 h, and then the solution was filtered using a 150 mesh screen to obtain the remaining solid. (4) The obtained solid was dried at 80 °C under vacuum until the weight of the solid was constant and the mass was recorded as *m*_n_. (5) The mass residual rate was calculated according to eqn (1).

### 2.11. Test of the SHA content in the EA

**Fig. 3:**
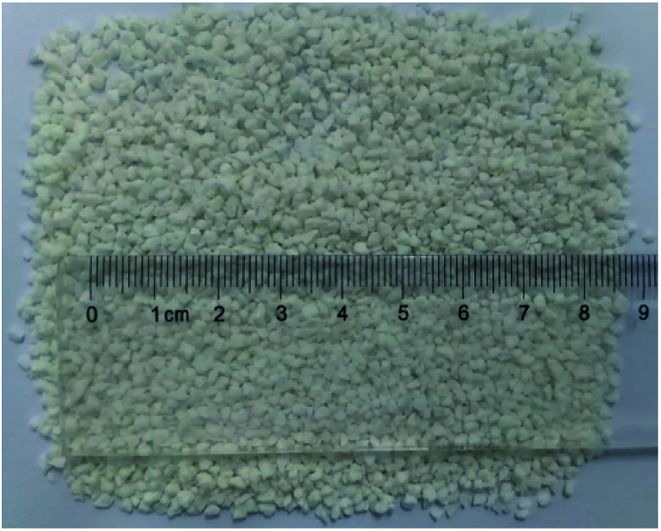
Appearance and size distribution of the EA.

As shown in Fig. 3, the EA was a particulate solid with a particle size of 10–20 mesh. The EA structure was divided into three layers, with the innermost layer being SHA and the outermost layer being the HTRCM, and the AP between them. The function of the HTRCM was to isolate the SHA, preventing the SHA from reacting in the near-well area. When the EA entered the far end of the fracture, the H^+^ ion concentration in the carrying fluid decreased and the encapsulating layer structure was destroyed, releasing the SHA to etch the fracture surface. Therein, the SHA content determined the etching performance on the fracture surface at the far end. The SHA content in the EA then was determined using a titration method.

The determination method was as follows: (1) an accurate amount (*M*_1_) of NaOH was weighed and then dissolved in deionized water (50 mL). The solution was transferred to a volumetric flask with 50 mL capacity and made up to 50 mL with sodium hydroxide solution. (2) A small amount of SHA was added into 20 mL of the sodium hydroxide solution prepared in step (1) and reacted for 20 min. The product from step (2) was titrated against hydrochloric acid with a concentration, *c*_1_, of about 0.25 mol L^−1^ until the pH value was 7, with the volume of hydrochloric acid consumed recorded as *V*_1_. (3) EA with the same mass as the SHA used in step (2) was added slowly into another 20 mL of sodium hydroxide solution and reacted for 20 min. After adding one drop of methyl orange, the product from step (3) was titrated against hydrochloric acid with a concentration of about 0.25 mol L^−1^ until the light red solution turned colorless, with the volume of hydrochloric acid consumed recorded as *V*_2_. The SHA content of the EA was calculated with eqn (2).2
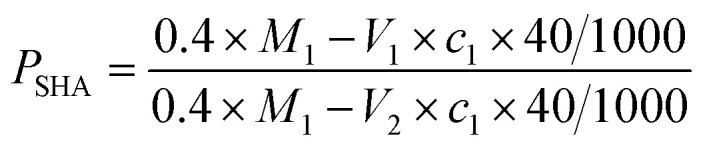


### 2.12. Etching performance tests

The etching performance by the HCl and the EA on the rock was using the following procedure: (1) 12 rock blocks were polished to produce a smooth surface. The surface areas of those rocks were approximately equal, and all rock blocks were put in a drying oven for two days. (2) Two hydrochloric acid solutions of 500 mL with a concentration of about 15% were prepared, to one of which was added a certain amount of EA, so that the EA mass fraction reached 5%. (3) All acid solutions were put in 12 reaction vessels follow the mixing ratio of 10 mL : 1 g with the rock blocks. Six pieces of rock block were exposed to the two acid solutions and each was numbered, and the reaction time recorded. Then the acid solutions and rock blocks were placed in a water bath to maintain the temperature at 90 °C while recording the reaction time. The rock blocks were taken out after reaction times of 10 min, 20 min, 30 min, 40 min, 50 min, 60 min, 120 min, and 180 min, after which they were washed, dried and measured. The residual acid concentrations were determined by titration and the rocks were weighed and their weights recorded. The detailed experimental parameters are shown in Table 1.

**Table 1:** Parameter of etching performance tests

Acid formulas	Rock number	Test time (min)	Reaction temperature (°C)
15% HCl	A1	10	90
	A2	20	
	A3	30	
	A4	40	
	A5	50	
	A6	60	
	A7	120	
	A8	180	
15% HCl + EA (*ω*_EA_ = 5%)	B1	10	
	B2	20	
	B3	30	
	B4	40	
	B5	50	
	B6	60	
	B7	120	
	B8	180	

### 2.13. Etched fracture conductivity test of the EA

**Fig. 4:**
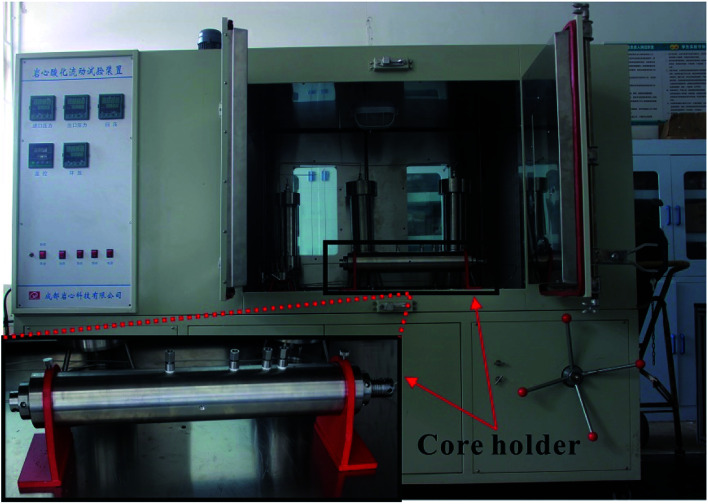
The acid-etched fracture conductivity equipment and the core holder.

Acid-etched fracture conductivity is one of the most important evaluation criteria for the acid fracturing effect.^27,28^ The acid-etched fracture conductivity test procedure (the theoretical analysis of the experiment refers to the work by Asadollahpour *et al.*^27^) was performed using the acid-etched fracture conductivity equipment (Fig. 4) produced by Chengdu Core Technology Co., Ltd in Sichuan Province to evaluate the performance of the EA. The experimental steps were as follows: (1) hydrochloric acid with concentrations of 5%, 10%, 15%, and 20% was prepared in duplicate, and to one of these was added an appropriate amount of EA to make *ω*_CCRSA_ = 5%. The acid formula is shown in Table 2. (2) A total of 16 rock plates with dimensions of 15 × 10 × 2 cm were manufactured and numbered 1–8. Eight pairs of rock plates were placed into the core holder. (3) The core holder was connected, the holder heating system was opened and the temperature was set to 120 °C. (4) A standard brine solution and the acid system were poured into the corresponding liquid tanks, the heating jacket was turned on and the temperature was set to 120 °C. (5) After the acid tank and core holder were heated to 120 °C, the acid etching step was performed with an acid injection displacement of 0.5 L min^−1^ and an acid injection time of 30 min. The outlet residual acid was neutralized by NaOH solution. (6) After exposure, the rock was taken out and the residual acid on the rock plate surface was washed away. A 3D scan of the surface was performed to obtain the surface morphology of the rock plates after acid etching. (7) After laser scanning, the eight pairs of rock plates were put into the core holder again to test the injection pressure difference and discharge under closing pressures of 5, 10, 20, 30, 40, and 50 MPa (that is, the normal stress of each pair of rock plates). The conductivity was calculated using eqn (3).3
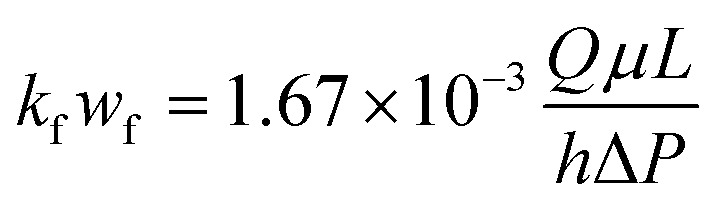
where *k*_f_*w*_f_ refers to the acid-etched fracture conductivity, μm^2^ cm; *Q* refers to the acid injection displacement, mL min^−1^; *μ* refers to the acid viscosity, mPa s; *L* refers to the rock plate length, cm; *h* refers to the rock plate width, cm; and Δ*P* refers to the pressure difference between the ends of the rock plate.

**Table 2:** Acid compositions during the conductivity tests

Number	1	2	3	4
Acid composition	5% HCl	10% HCl	15% HCl	20% HCl
Number	5	6	7	8
Acid composition	5% HCl + EA	10% HCl + EA	15% HCl + EA	20% HCl + EA
	*ω*_CCRSA_ = 5%	*ω*_CCRSA_ = 5%	*ω*_CCRSA_ = 5%	*ω*_CCRSA_ = 5%

## 3. Results and discussion

### 3.1. ESEM and energy spectrum analysis of the EA

**Fig. 5:**
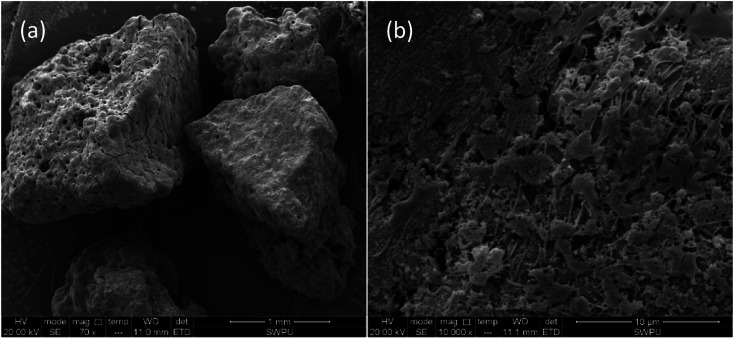
Microscopic appearance of the EA ((a) magnified 70 times; (b) magnified 10 000 times).

The ESEM test results for the EA are summarized in Fig. 5. EA particles are shown in Fig. 5a. In the upper left corner of the photograph, a number of small holes with different sizes between 10 and 30 μm in diameter are evident on the surfaces of the EA particles. These holes resulted from uneven spraying during encapsulation. Most of the EA particles had dense, non-permeable encapsulation layers on the surface and hence the HTRCM achieved effective encapsulation for the SHA. The surface morphology of the EA, magnified 10 000 times, is shown in Fig. 5b. As can be observed from Fig. 5b, the large number of fibrous polymer materials further confirmed that the surface of the EA was successfully encapsulated by the HTRCM.

**Fig. 6:**
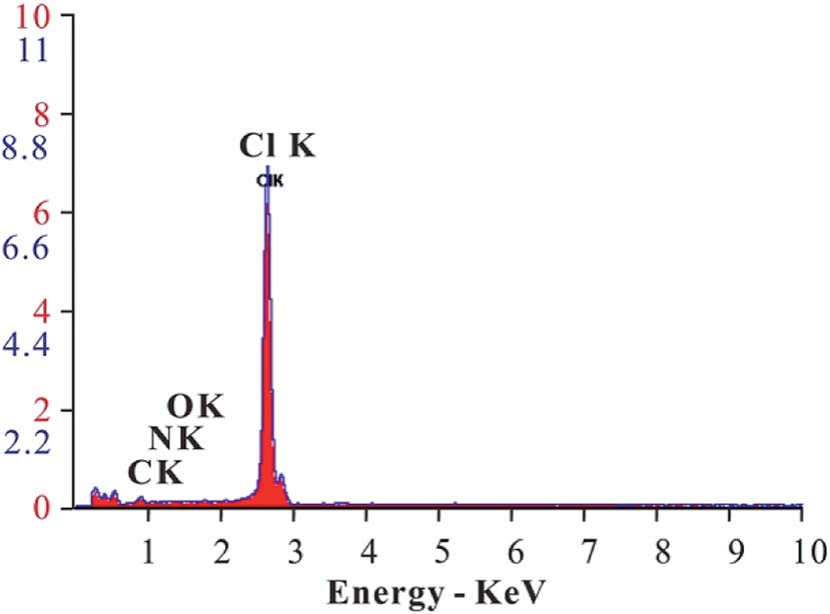
Energy spectrum analysis plot for the EA.

The energy spectrum analysis curve of the EA is shown in Fig. 6. From the analysis results, the EA mainly consisted of C, N, O, Cl and other elements. The C, N, and O shown in the energy spectrum analysis results were from the HTRCM and the Cl was from the SHA. It was confirmed that the SHA was effectively encapsulated by the HTRCM. As energy spectrum analysis cannot test the H element content, it cannot be simply inferred that the EA did not contain the element H. Instead, it was inferred that the H element content was not too low from the raw material synthesizing the HTRCM and the elemental composition of encapsulated SHA.

### 3.2. Thermogravimetric analysis of the SHA

**Fig. 7:**
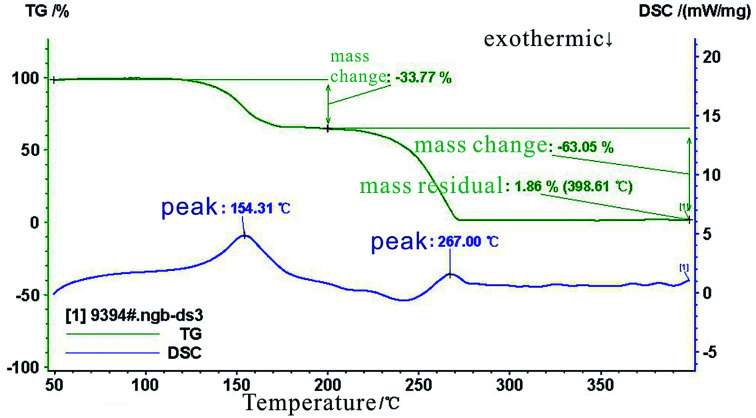
Change of solid acid mass with temperature.

The results of the thermogravimetric (TG) analysis and from the differential scanning calorimeter (DSC) tests are shown in Fig. 7. These results show that the mass of the SHA remained unchanged when the temperature was increased from 45 °C to 135 °C. The SHA began to decompose and the mass decreased when the temperature exceeded 135 °C. The SHA had decomposed completely at 267 °C. From the DSC results, when the temperature increased to 154.31 °C, an exothermic peak appeared, the SHA decomposition rate decreased, and the residual mass tended to be stable. At 267 °C, a peak appeared in the DSC curve again, and the SHA almost decomposed completely. These results show that SHA retained its thermal stability below 135 °C.

### 3.3. FTIR results

**Fig. 8:**
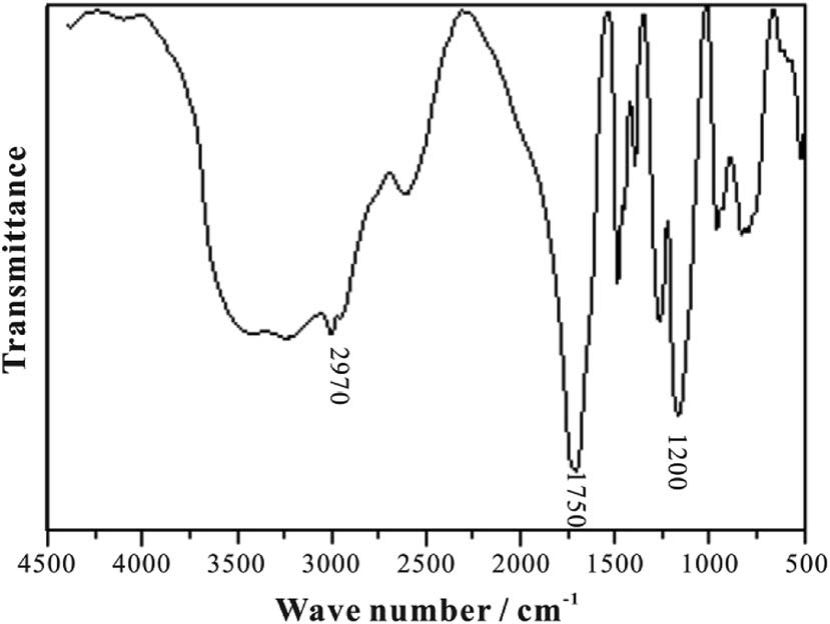
The infrared spectrum of HTRCM.

The infrared spectrum of HTRCM is shown in Fig. 8. The amide group N–H absorption peak and the carboxylic group O–H absorption peak appeared at about 3200–3500 cm^−1^, the absorption peak of the main chain structure –CH_2_– appeared at about 2970 cm^−1^, the absorption peak of C=O in AL and AC appeared at about 1750 cm^−1^, and the absorption peak of C–O–C in AAP appeared at about 1200 cm^−1^. Hence, it can be inferred that AL, AC, DL, and AAP all linked to HTRCM successfully.

### 3.4. NMR results

**Fig. 9:**
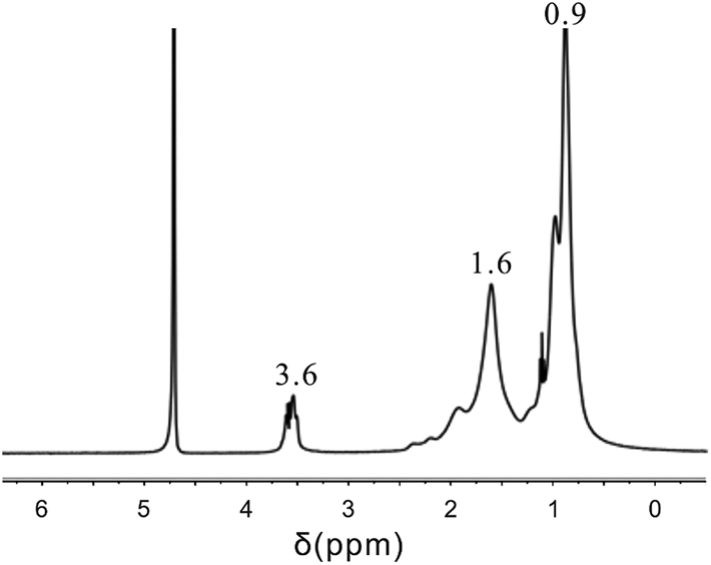
^1^H NMR spectrum.

It can be seen from the ^1^H NMR spectrum given in Fig. 9 that the NMR peak of H atoms from the polyether in AAP appeared at about 3.6 ppm, the NMR peak of H atoms from the –CH_2_– structure in the DL appeared at about 1.6 ppm, and the NMR peak of H atoms from the carboxylic ester in AC and the alkyl in AAP appeared at about 0.9 ppm. The HTRCM contained the structures from the AL, AC, PL, and AAP; that is, the HTRCM exhibited the designed target structure.

### 3.5. Mechanical performance of the HTRCM

**Fig. 10:**
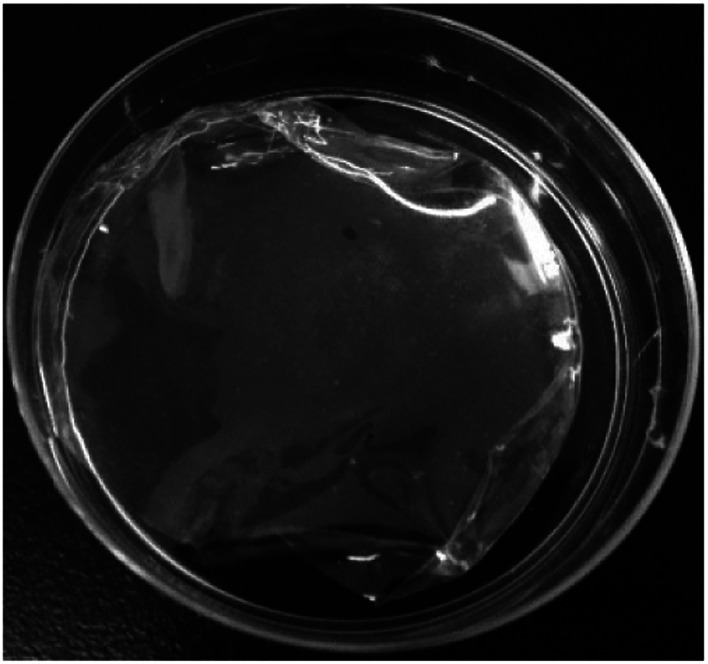
The appearance of the HTRCM film.

Fig. 10 demonstrates the appearance of the HTRCM after film formation. The film made by HTRCM was complete on the surface and had a certain toughness.

**Fig. 11:**
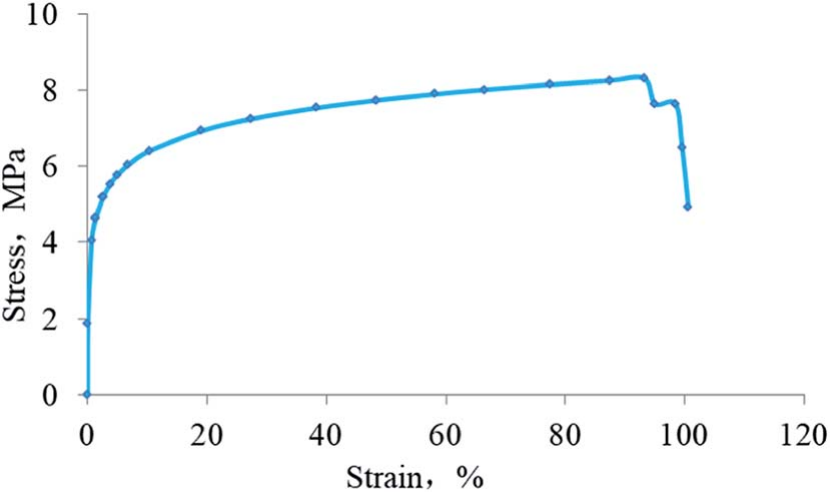
Stress–strain curve of HTRCM.

The stress–strain curve of the HTRCM is shown in Fig. 11. The maximum tensile stress of the HTRCM was 8.31 MPa and the strain reached 91%. The test results showed that HTRCM not only had a certain strength but also had suitable deformation characteristics and impact resistance.

### 3.6. Dissolution of the HTRCM

**Fig. 12:**
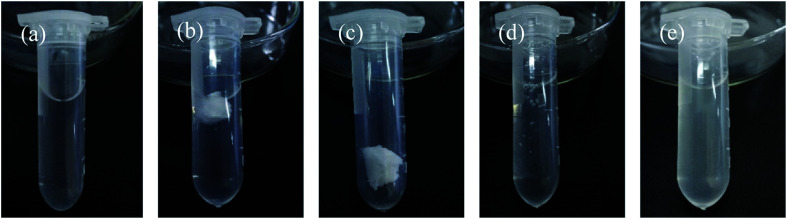
Dissolution of the HTRCM in different solvents (the solubility is determined using eqn (1) where *R* > 95% is insoluble, 20% < *R* ≤ 95% is partially dissolved, 5% < *R* ≤ 20% is easily soluble, and 0 ≤ *R* ≤ 5% is highly soluble). (a) AE, (b) EAC, (c) DE, (d) deionized water, and (e) 0.01 g mL^−1^ NaOH solution.

The dissolution behavior of the HTRCM in different solvents at room temperature is illustrated in Fig. 12. As can be seen from the figure, HTRCM did not dissolve in EAC or DE but had good solubility in AE, deionized water, and NaOH solution (0.01 g mL^−1^). The test results showed that the HTRCM will dissolve completely in alkaline and neutral solvents, releasing the internal encapsulated SHA.

**Fig. 13:**
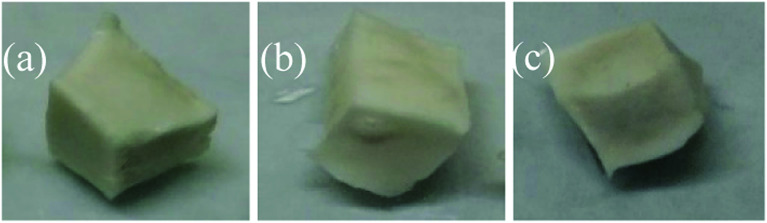
Dissolution of the HTRCM in concentrated hydrochloric acid and sodium hydroxide solution at 135 °C. (a) Before the dissolution test in concentrated HCl solution. (b) After the dissolution test in concentrated HCl solution. (c) Before the dissolution test in NaOH solution.

The dissolution of the HTRCM in the acid and alkali solutions at higher temperature (135 °C) is illustrated in Fig. 13. It can be observed from the test results that the HTRCM hardly dissolved in the concentrated hydrochloric acid and retained a very obvious faceted surface structure but dissolved completely in the sodium hydroxide solution. The results show that the HTRCM is insoluble in high-concentration acid solution (37%) and can be used to encapsulate SHA. It will release the encapsulated SHA with the consumption of H^+^ ions.

### 3.7. Effect of the H^+^ ion concentration in the carrying fluid on the release of the EA

**Fig. 14:**
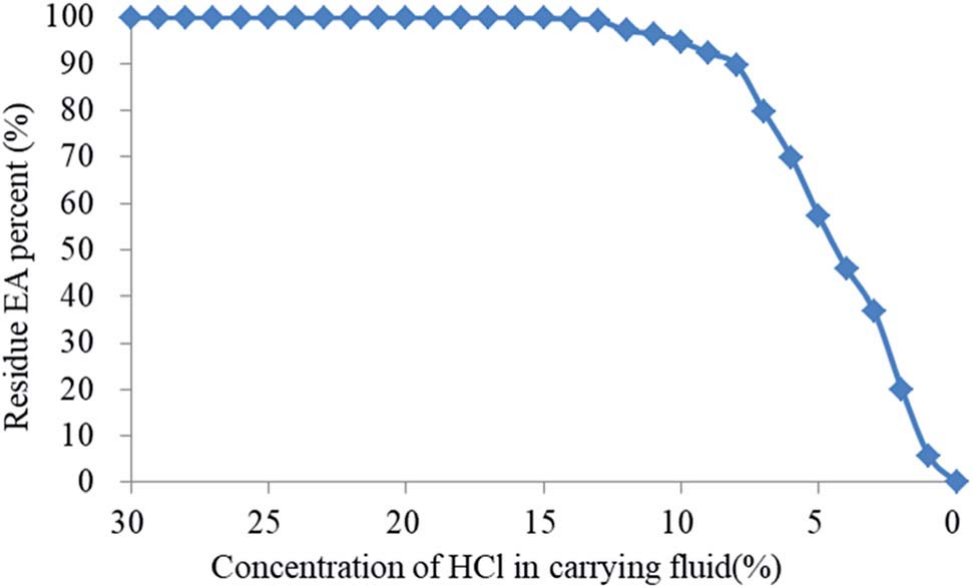
Effect of different H^+^ concentrations in the carrying fluid on the residual rate of the EA.

The effects of different H^+^ concentrations in the carrying fluid on the residual rate of EA are illustrated in Fig. 14. When the HCl concentration in the carrying liquid fell from 30% to 14%, the mass residual rate of the EA was still around 100% after 1 hour at 135 °C, which indicated that the SHA had not been released. When the H^+^ concentration of the carrying fluid decreased from 14% to 8%, the residual mass rate of the EA decreased slowly. However, when the H^+^ concentration was below 8%, the residual mass rate of the EA decreased quickly. Compared with the existing encapsulated acids,^18–22^ the encapsulating material in this paper is completely dissolved in neutral solution, avoiding damage to the reservoir by the residue.

As observed, the acid concentration had a great effect on the SHA release. The H^+^ ion concentration was high near the wellbore and the structure of the HTRCM was complete, which effectively isolated the SHA. However, when the EA had been transported to the far end zone of the artificial fracture, the H^+^ ion concentration in the carrying liquid is gradually consumed, resulting in a decrease in the H^+^ ion concentration. As the structure of the HTRCM is destroyed, releasing SHA, ionization produces new H^+^ ions, which restores the H^+^ ion concentration of the carrying fluid, resulting in a decrease in the release rate of the SHA. If the concentration of H^+^ ions decreases, the EA releases the SHA to restore the H^+^ ion concentration. Nevertheless, the acid–rock reaction consumes the H^+^ ions, resulting in the decrease of the H^+^ ion concentration. The above cyclical process increases the etching length of acid fracturing, as shown in Fig. 1.

### 3.8. Effects of temperature on EA release

**Fig. 15:**
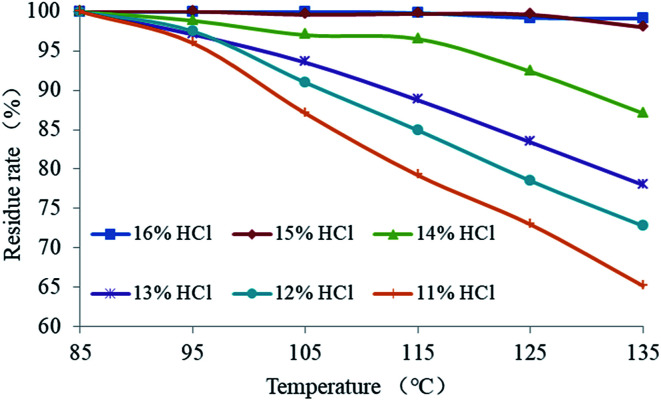
Effect of temperature on EA release.

The effect of temperature on the EA release rate is shown in Fig. 15. It demonstrates that the residual mass rate of the EA decreased with the increase of temperature when the hydrochloric acid concentration was 11–14%. The degree of decrease in the residual rate was inversely proportional to the hydrochloric acid concentration. The mass residual rate of the EA in 11% hydrochloric acid decreased to 65% at 135 °C. When the hydrochloric acid concentration was 15% or more, the mass residual rate of the EA was almost unchanged with the increase of temperature. As a result, the critical hydrochloric acid concentration of the EA release was 15%. The results demonstrated that the HTRCM has good acid-resistance performance in 15% HCl solution at 135 °C and that the EA still has good acid-resistance performance even in 11% HCl solution at 95 °C, which indicates that the critical release temperature of the EA is 95 °C (hydrochloric acid concentration ≤ 14%).

**Fig. 16:**
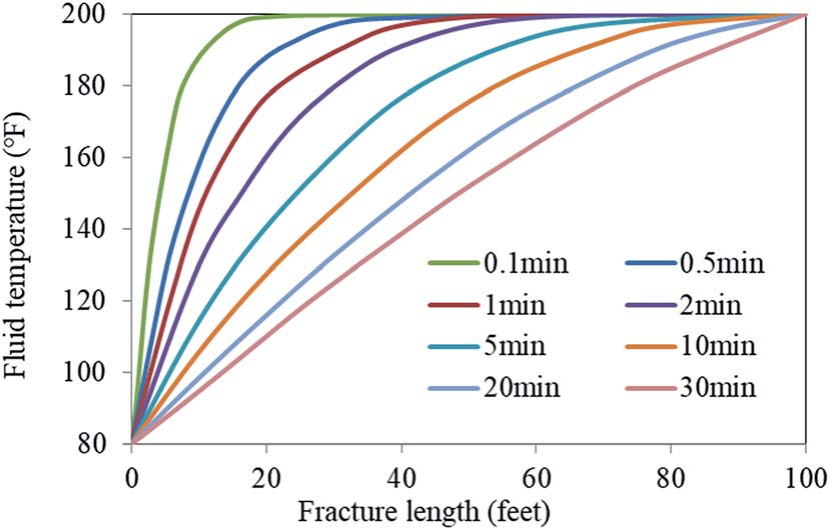
Temperature distribution from the well along the fracture.^29^

The temperature in the hydraulic fractures is shown in Fig. 16. As can be observed, the temperature was lower near the well and gradually increased along the fracture.^29^ The temperature at the leading fracture edge was equal to the initial reservoir temperature. According to the EA release under different temperature conditions shown in Fig. 15, the fracture temperature field can be used to further control the release of the EA. Owing to the lower temperature near the well, the SHA was hardly released. When the EA migrated to the middle area of the hydraulic fracture, the reaction between the acid and the rock caused a decrease in the H^+^ ion concentration in the carrying liquid. As a result, the EA began to release the SHA, which entered the cycle process shown in Fig. 1. Because of the lower temperature, the SHA did not release completely, which allowed the EA to migrate to the far end of the fractures and improved the etching length.

### 3.9. The effective content of the EA

Test results for the effective content of the SHA test and the SHA content in the EA as calculated by eqn (2) are summarized in Table 3. As can be observed, the mass content of SHA in the EA was nearly 85%, and encapsulating material accounted for about 15%. The mass content of SHA was high, which enabled it to achieve better etching effects.

**Table 3:** Test results of the effective content

Group	Mass of NaOH *M*_1_ (g)	Hydrochloric acid concentration, *c*_1_ (mol L^−1^)	Hydrochloric acid volume of titration SHA, *V*_1_ (mL)	Hydrochloric acid volume of titration EA, *V*_2_ (mL)	SHA content, *P*_SHA_
1	2.501	0.25	32.45	20.21	84.67%
2	2.502		32.42	20.35	84.86%
3	2.501		32.39	20.43	84.98%

### 3.10. Etching performance of the EA

**Fig. 17:**
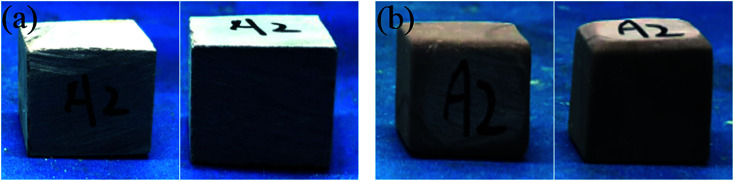
A2 rock appearance. (a) Rock sample before etching in HCl and (b) rock sample after etching in HCl.

**Fig. 18:**
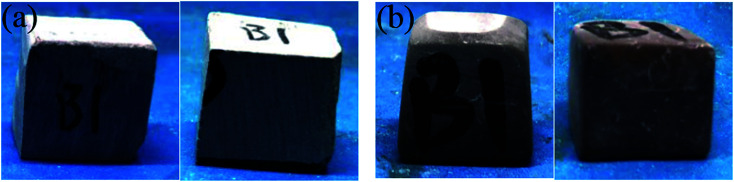
B1 rock appearance. (a) Rock sample before etching in HCl + EA and (b) rock sample after etching in HCl + EA.

The appearance of the rock samples before and after etching by HCl and HCl + EA is shown in Fig. 17 and 18, respectively. The rock block size and acid concentration before and after etching are presented in Table 4. As shown in Fig. 17 and 18, the surface of all rock block was smoother after etching than was the case before etching, but the extent of etching was not equal at different positions on the rock surface and the rock samples had a trapezoidal shape after etching.

**Table 4:** Rock mass and acid concentration before and after etching

Core number	Before etching		After etching		Rock consumption mass (g)
	Rock mass (g)	Acid concentration (%)	Rock mass (g)	Acid concentration (%)	
A1	10.65	15	9.51	13.97	1.14
A2	10.16	15	7.98	12.84	2.17
A3	10.34	15	7.67	12.02	2.67
A4	10.51	15	6.82	11.51	3.69
A5	9.36	15	5.25	10.49	4.11
A6	9.93	15	5.19	10.26	4.74
A7	10.37	15	4.85	6.42	5.52
A8	9.83	15	3.92	4.21	5.91
B1	9.02	15	7.43	15.25	1.59
B2	9.20	15	6.29	14.70	2.91
B3	11.97	15	7.71	13.89	4.26
B4	8.32	15	3.56	13.24	4.76
B5	10.85	15	5.20	11.67	5.64
B6	10.27	15	3.53	11.19	6.73
B7	10.63	15	3.34	8.73	7.29
B8	10.69	15	1.76	8.55	8.92

**Fig. 19:**
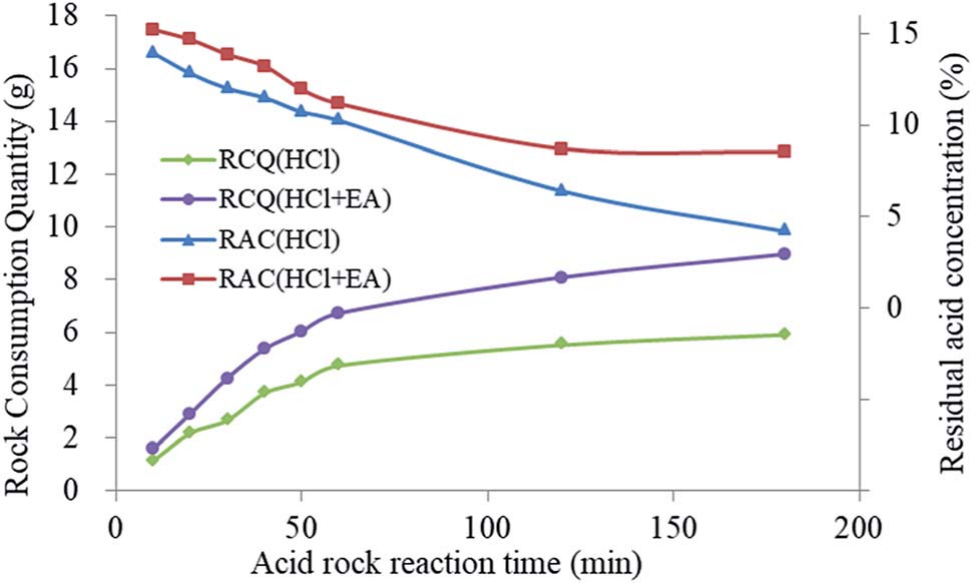
Rock consumption quantity (RCQ) and residual acid concentration (RAC) for different reaction times.

From the etching test results shown in Table 4, the degree of rock block consumption increased with the increase of the etching time in HCl solution. Nevertheless, when the reaction time exceeded 60 min, the consumed rock mass tended to be stable as the hydrochloric acid concentration had decreased, resulting in an inability to consume more rock. When HCl + EA solution was used, the EA could release SHA to restore the H^+^ ion concentration after H^+^ consumption. Thus, more rock was consumed. As shown in Fig. 19, when the etching time was less than 60 min, the rock mass consumption increased with an associated rapid decrease in acid concentration using HCl + EA. When the etching time exceeded 60 min, the residual acid concentration was stable at nearly 8% and the quantity of rock consumption continued to increase.

The results of the acid–rock consumption tests further confirmed the differences in the dynamic change in H^+^ ion concentration between the two acid mixtures. The concentration of H^+^ ions in the carrying fluid decreased owing to the chemical process between the rock and the acid. The HTRCM structure was destroyed owing to the dual control by H^+^ ions and temperature, releasing the encapsulated SHA to restore the H^+^ ion concentration in the fluid and maintain the concentration of the acid. The H^+^ ion concentration change was in a state of dynamic equilibrium, as shown in Fig. 1.

### 3.11. Acid-etched fracture conductivity for the EA

**Fig. 20:**
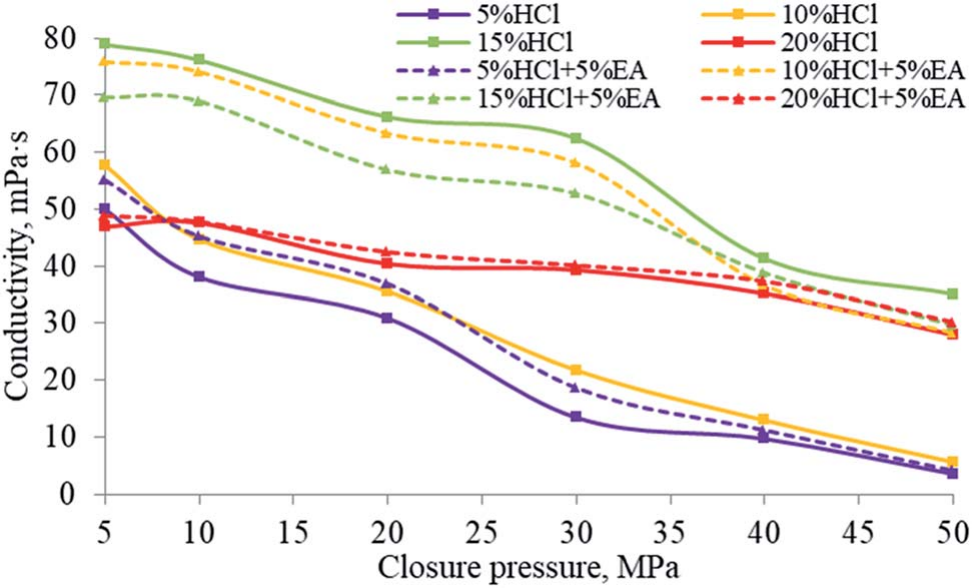
Acid persistence for different hydrochloric acid concentrations and for hydrochloric acid mixed with the EA.

The degree to which the acid etching capability persists along the fracture path under different hydrochloric acid concentrations and for hydrochloric acid mixed with EA is shown in Fig. 20. At higher closing pressures, the conductivity of different acid mixtures was decreased. Using different concentrations of hydrochloric acid for etching, the measured conductivity first increased and then decreased. This was because when the hydrochloric acid concentration was lower than 15%, the amount of rock mineral consumed by the acid increased and therefore the ability of the acid to permeate along the fracture faces increased with the increase in acid concentration. However, when the acid concentration exceeded 15%, excessive etching of the rock took place, causing the fracture faces to close, which resulted in a decrease in the capability of the injected acid to permeate along the fracture faces in the rock strata. It can be inferred from Fig. 14 that the EA completely released its SHA in 5% and 10% HCl solution, thereby maintaining the H^+^ ion concentration in the solution, and therefore the conductivity when using (5% HCl + 5% EA) was higher than that when using only 5% HCl, and was similar to the performance when using 10% HCl. The conductivity when using (10% HCl + 5% EA) was much higher than that when using straight 10% HCl. In addition, it can be inferred from the results shown in Fig. 20 that the EA released hardly any SHA in 15% HCl. However, once a proportion of the H^+^ ions had been consumed, that promoted the EA to release SHA to restore the H^+^ ion concentration in the solution. Excessive etching was evident when using (15% HCl + 5% EA), which resulted in a lower acid persistence than when using 15% HCl. Compared with 15% HCl, more H^+^ ions must be consumed from 20% HCl before the release of the SHA from the EA is triggered. During these tests, owing to the shorter cores employed, the amount of residual H^+^ ions did not reach the critical release concentration for the EA. As the release of SHA from the EA was less, the conductivity using 20% HCl was close to that using 20% HCl + 5% EA.

**Fig. 21:**
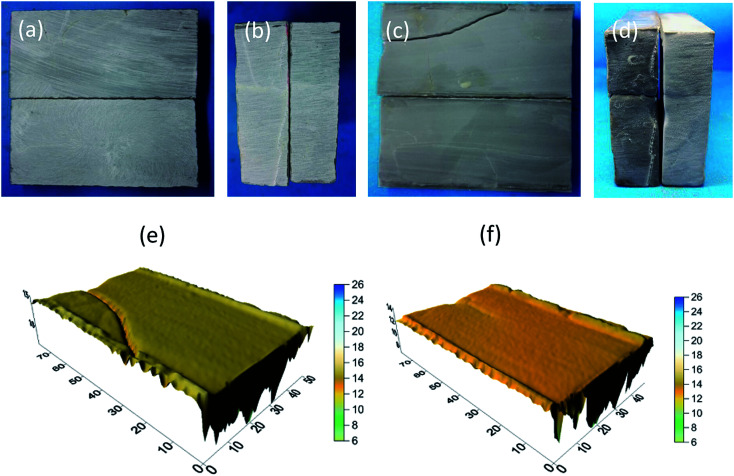
10% HCl conductivity experimental results. (a) Morphology of fracture surface before acid etching; (b) morphology of fracture inlet before acid etching; (c) morphology of fracture surface after acid etching; (d) morphology of fracture inlet after acid etching; and (e and f) 3D scanning morphology of fracture surface after acid etching.

**Fig. 22:**
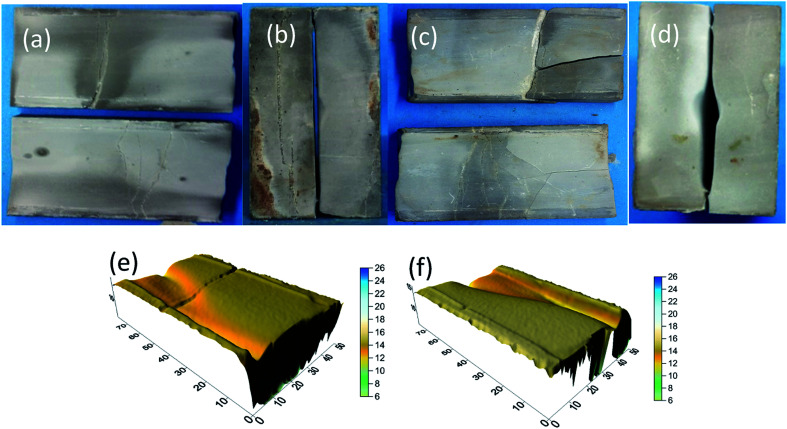
10% HCl + 5% EA conductivity experimental results. (a) Morphology of fracture surface before acid etching; (b) morphology of fracture inlet before acid etching; (c) morphology of fracture surface after acid etching; (d) morphology of fracture inlet after acid etching; and (e), (f) 3D scanning morphology of fracture surface after acid etching.

The findings of the tests for 10% HCl and 10% HCl + 5% EA conductivity are shown in Fig. 21 and 22. Before etching (Fig. 21a and 22a), the sample surfaces were smooth without obvious grooves and the fracture width formed by the combination of the two rock plates was narrow (Fig. 21b and 22b). After acid etching the rock plate, the acid reacted with the cement in the rock plate, causing the rock plate to break (Fig. 21c and 22c). After etching (Fig. 21d and 22d), the fracture width formed by the combination of the two rock plates was significantly larger than that before the etching.

From the results of laser scanning analysis of the rock plates after etching (Fig. 21e, f and 22e, f), etching grooves appeared on the etched surfaces, which increased the fracture conductivity. However, compared with those produced by the 10% HCl, the etching grooves of 10% HCl + 5% EA were deeper and, as a consequence, the conductivity was higher.

## 4. Conclusions

In this study, a novel EA has been described that is comprehensively controlled by hydrogen ion concentration and temperature. The described procedures included the preparation of the solid acid SHA, the preparation of the encapsulation material HTRCM for controllable release, and the encapsulation of the solid acid.

ESEM and energy spectrum analysis confirmed that the surface of the EA was successfully encapsulated by the encapsulation material. Thermal stability tests conducted on the solid acid demonstrated that the solid acid can maintain its stability below 135 °C. FTIR and NMR tests demonstrated that the encapsulation material achieved its intended functionality. Mechanical tests of the encapsulation material demonstrated that the material had good mechanical strength, strong impact resistance, and adequate abrasion resistance. Dissolution tests demonstrated that the encapsulation material had the required characteristics of acid resistance and good encapsulation performance. Experiments to investigate the release behavior, effective solid acid content, etching ability, and fracture conductivity demonstrated that the controlled release of the EA prepared using the encapsulation approach was highly effective in maintaining the acid concentration, its etching capability and the controllable release of H^+^ ions.

Compared with existing encapsulated acids, the encapsulating material in this paper is completely dissolved in neutral solution, thus avoiding reservoir damage by the residue.

## Nomenclature

EA: Encapsulated acid
ESEM: Environmental scanning electron microscope
TGA: Thermogravimetric analysis
FTIR: Fourier transform infrared spectroscopy
NMR: Nuclear magnetic resonance
HTRCM: H^+^ ion-temperature responsive coating material
SHA: Solid hydrochloric acid
AL: Acrylamide monomers
AC: *tert*-Butyl allyl carboxylate monomers
DL: Diallylamine monomers
AAP: Allyl alkyl polyether monomers
AE: Absolute ethanol
AP: Anion polyacrylamide
EAC: Ethyl acetate
DE: Dichloromethane

## Conflicts of interest

The authors declare no competing financial interest.

## Supplementary Material
